# Keratoconus in Thai population – a cross-sectional hospital-based study

**DOI:** 10.1515/abm-2022-0035

**Published:** 2023-08-01

**Authors:** Yonrawee Piyacomn, Ngamjit Kasetsuwan, Vilavun Puangsricharern, Usanee Reinprayoon, Vannarut Satitpitakul, Patchima Chantaren

**Affiliations:** Department of Ophthalmology, Excellence Center for Cornea and Limbal Stem Cell Transplantation, King Chulalongkorn Memorial Hospital, Faculty of Medicine, Chulalongkorn University, Bangkok 10330, Thailand

**Keywords:** collagen crosslinking, corneal ectasia, intracorneal ring, keratoconus, refractive surgery

## Abstract

**Background:**

Studies in the epidemiology of keratoconus are limited in Southeast Asia. A study on the prevalence and characteristics of keratoconus in the Thai population could give a general idea of its impact.

**Objectives:**

To study keratoconus prevalence in patients seeking refractive surgery and analyze the characteristics of keratoconus.

**Methods:**

Medical records from April 2015 to August 2018 were retrospectively reviewed. Keratoconus and keratoconus suspect prevalence in patients seeking laser vision correction were calculated. The characteristics of keratoconus patients were reviewed. The Amsler–Krumeich classification was used to determine the stages. Topographically, the types of cones were categorized into oval, nipple, pellucid marginal degeneration (PMD)-like, and astigmatic types.

**Results:**

Keratoconus and keratoconus suspect prevalence were 1.66% and 0.68%, respectively. Out of the affected patients, 73.8% were male. The mean age at diagnosis was 25.25 ± 8.35 years. The presenting symptoms were blurred vision (87%) and itching (47%). Stage 1 was predominant, found in 39% of patients (followed by stages 2, 4, and 3, respectively). Ocular findings comprised the Munson sign (14.63%), the Rizutti sign (6.94%), Fleischer ring (28.14%), Vogt striae (24.95%), corneal scar (8.63%), prominent corneal nerve (2.81%), and corneal staining (7.69%). Mean uncorrected visual acuity (logarithm of the minimum angle of resolution [logMAR]) was 0.88 ± 0.64. Mean corrected visual acuity (logMAR) was 0.40 ± 0.49. Mean Q-value was −0.92 ± 0.63. The thinnest pachymetry was 459.39 ± 56.96 microns. The mean keratometry was 49.7 ± 6.64 diopters. Topographically, the types of cones were oval (57%), astigmatic (33%), PMD-like (5%), and nipple type (4%).

**Conclusions:**

Keratoconus prevalence among Thai patients seeking refractive surgery was 1.66%. Most patients were male and presented with the disease at a mild bilateral stage in their second decade of life.

Keratoconus is a slowly progressive corneal ectatic disease. The noteworthy characteristics of the disease are thinning and protruding of central or paracentral cornea. It causes myopia, irregular astigmatism, and a reduction in visual acuity [1]. Early detection is important not only to slow or halt disease progression but also to avoid unpredictable outcomes before undergoing any refractive surgery. Late diagnosis leads to severe manifestations, for example, corneal hydrops and scarring. It usually affects both eyes. The prevalence varies among several factors; for example, ethnicity, geographical area, and presence of concomitant diseases (e.g., atopy) [1]. The reported prevalence rates vary from 0.02 to 3333 cases per 100,000 population [2, 3, 4, 5, 6, 7, 8, 9, 10, 11, 12]. Reports from the Middle East and India are higher than those from Russia, the USA, and the UK [3, 4, 5, 7, 8, 10, 11]. Therefore, geographical distribution and ethnicity are significant risk factors influencing the prevalence and the incidence.

There are some reported cases that imply the genetic etiology might play a role. Evidences include familial inheritance, concordance between monozygotic twins, and the association of keratoconus to other known genetic disorders such as Leber congenital amaurosis, Down syndrome, Marfan syndrome, or Ehlers–Danlos syndrome [11, 13, 14].

Studies in the epidemiology of keratoconus are limited in Southeast Asia. Furthermore, there is an increasing number of patients seeking corneal refractive surgery or laser vision correction. As a result, there is a necessity to study the prevalence of keratoconus. To our knowledge, this is the first study to state the prevalence and also the general characteristics of keratoconus in the Thai population.

## Methods

This observational study included all patients who requested refractive surgery at Chula Refractive Surgery Center, Bangkok, Thailand, from April 2015 to July 2018. We reviewed all patients who had come and registered at Chula Refractive Surgery Center by searching in the registration book. Normally all patients who requested laser vision correction would undergo the corneal topography from a Pentacam device (Oculus, Inc.). All patients from the registration book matched the topography database. We calculated the prevalence of keratoconus and keratoconus suspect from these patients’ data. The other part of our study was that we evaluated the clinical characteristics of patients with keratoconus, both those who first asked for refractive surgery at Chula Refractive Surgery Center and those who had already been diagnosed with the disease by ophthalmologists in general eye clinics or the cornea clinic at King Chulalongkorn Memorial Hospital, Bangkok, Thailand from April 2015 to July 2018. We extracted the data from reviewing all patient records and corneal topography from the corneal tomography device. We also avoided missing data by retrieving the medical records from both the computer database and the corneal topography device for reviewing. To avoid selection bias, we excluded the patients who did not have corneal topography results.

The study followed the tenets of the Declaration of Helsinki and was approved by the Institutional Review Board, Faculty of Medicine, Chulalongkorn University (Certificate of approval No. 1124/2018). Our study also followed the REporting of studies Conducted using Observational Routinely-collected health Data (RECORD) and Strengthening the Reporting of Observational Studies in Epidemiology (STROBE) statements [15, 16]. The recorded patient characteristics were age, sex, and laterality of the eye. The patients were asked how they had previously corrected their refractive errors, for example, with glasses or contact lenses. The ocular examination included visual acuity measurement, refraction, corneal tomography evaluation using a corneal tomography device (Pentacam; Oculus, Inc.), slit lamp biomicroscopy, and fundus examination. Visual acuity was recorded and converted into logarithm of the minimum angle of resolution (logMAR) units. The Munson sign and the Rizutti sign were noted. Slit lamp biomicroscopy was used to demonstrate Fleischer ring, Vogt striae, corneal scar, prominent corneal nerve, and corneal staining. For ocular surface staining, we used fluorescein dye, a mildly invasive stain that marks tear film and defects in the corneal and conjunctival epithelium. The Amsler–Krumeich classification was used to classify the keratoconus stage. Corneal topography was also used to classify the cone type in each keratoconic eye. If there were more than 1 visit for each patient, there would be more than 1 corneal topography result. Only the first eligible corneal topography results were analyzed. We excluded the topography results corresponding to examinations in which patients had not taken off their contact lenses (3 d for soft contact lenses and 7 d for rigid gas-permeable contact lenses) before undergoing imaging from the corneal topography device. These topography results were considered ineligible results.

The diagnosis of keratoconus was made using a Belin-Ambrósio enhanced ectasia display (BAD) (Pentacam). This helped evaluate anterior and posterior elevation, pachymetry, and keratometry. The parameters assessed by BAD have been described in previous studies [17, 18, 19]. The final D value derived from the display was used to classify the abnormality using a color code. There are 3 color codes: white (normal) when the final D value is <1.6 standard deviation (SD) from the population mean, yellow (suspicious) when the final D value is between 1.6 SD and 2.6 SD, and red (abnormal or pathologic) when the final D value is >2.6 SD.

Topographically, cones are classified into 4 types: oval, nipple, astigmatic, and pellucid marginal degeneration-like (PMD-like). The oval type was defined as having a Q-value between 0.23 and −1.00 with moderate corneal astigmatism. Nipple type was defined as having a Q-value >−1.00 with astigmatism <2.5 D. Astigmatic type was defined as having a Q-value >−1.00 and astigmatism >4.0 D. PMD-like was defined as having a Q-value <−0.23.

All data were collected in Microsoft Excel. Regarding data cleaning, we removed duplicate data by rechecking the patient's hospital number.

The demographic data, including gender, affected eye, presenting symptoms, and previous visual rehabilitation, were counted as numbers and percentages. The disease characteristics data, including disease staging, ocular signs, type of corneal topography, and any needs for corneal transplantation, were counted as numbers and percentages. Mean and SD were calculated for visual acuity, Q-value, thinnest pachymetry, and mean keratometry.

## Results

Of 1923 patients who asked for corneal refractive surgery from April 2015 to August 2018 at our center, 32 were diagnosed with keratoconus (1.66%), and 13 were diagnosed with keratoconus suspect (0.68%).

When we considered patients who first asked for laser vision correction and those who had been diagnosed with keratoconus from a general eye clinic or the cornea clinic, there were 271 patients with keratoconus at our center. The characteristics of keratoconus patients and keratoconic eyes included not only those of patients seeking refractive surgery but also those of patients from a general eye clinic or the cornea clinic. All keratoconus patients have at least 1 eligible corneal topography result.

The mean age at diagnosis was 25.25 years (SD, 8.35 years). The demographic data, the disease characteristics, and the visual parameters are shown in **Tables 1–3**, respectively.

**Table 1.** Demographic data

**Demographic data**	**Number of patients (percentage) n = 271**
**Gender**	
Male	200 (73.8)
Female	71 (26.2)
**Affected eye**	
Only right eye	5 (1.9%)
Only left eye	4 (1.5%)
Both eyes	262 (97%)
**Presenting symptoms**	
Itching	128 (47.2)
Known allergy†	48 (17.7)
Blurred vision or frequently needed new glasses prescription	237 (87.5%)
**Previous visual rehabilitation**	
Glasses	141 (52)
Contact lens‡	27 (10)
Both glasses and contact lens	16 (5.9)
None	87 (32.1)

† Either allergic conjunctivitis or rhinitis.

‡ Soft, soft toric, rigid gas-permeable, and Rose K lenses.

**Table 2.** Disease characteristics (totally 533 eyes in 271 patients)

**Disease characteristics**	**Number of eyes (percentage) n = 533 eyes**
**Amsler–Krumeich classification**	
Stage 1	206 (38.6)
Stage 2	169 (31.7)
Stage 3	36 (6.8)
Stage 4	122 (22.9)
**Signs**	
Munson sign	78 (14.8)
Rizutti sign	37 (6.9)
Fleischer ring	150 (28.1)
Vogt striae	133 (25)
Corneal scarring	46 (8.6)
Corneal staining	41 (7.7)
Prominent corneal nerve	15 (2.8)
**Corneal topography**	
Oval	305 (57.2)
Nipple	22 (4.1)
PMD-like	28 (5.3)
Astigmatic	178 (33.4)
**Management (ICRS or CXL)**	
ICRS	105 (19.7)
CXL	145 (27.2)
Both ICRS and CXL	65 (12.2)
**Corneal transplantation need†**	
Unilateral	12 (4.4)
Bilateral	103 (38)
No need	156 (57.6)

† Considered n = 271, either deep anterior lamellar keratoplasty or penetrating keratoplasty.

CXL, collagen crosslinking; ICRS, intracorneal ring segment insertion; PMD-like: pellucid marginal degeneration-like.

**Table 3.** Visual parameters (totally 533 eyes)

**Visual parameters**	**Mean ± SD (min–max)**
Uncorrected visual acuity (LogMAR)	0.88 ± 0.64 (−0.1 to 2.3)
Best-corrected visual acuity† (LogMAR)	0.40 ± 0.49 (−0.1 to 2.2)
Q-value	−0.92 ± 0.63 (−2.73 to 2.15)
Thinnest pachymetry (microns)	459.39 ± 56.96 (282–589)
Mean keratometry (diopter)	49.7 ± 6.64 (33.2–80.3)

† Best-corrected visual acuity refers to *measurement of the best vision correction that can be achieved using glasses or contact lenses*.

LogMAR, logarithm of the minimum angle of resolution; SD, standard deviation.

## Discussion

A literature review showed a higher prevalence of keratoconus in India, Lebanon, Iran, Saudi Arabia, and Israel (range: 2340–4000 cases/100,000 population) [2, 3, 12, 20, 21, 22]; and a lower prevalence in Korea, North America, Japan, Russia, Denmark, The Netherlands, and the United Kingdom (range: 0.2–265 cases/100,000 population) [5, 8, 10, 23, 24, 25].

Laser vision correction is generally contraindicated in keratoconus or keratoconus suspect patients because excimer laser ablation reduces the corneal thickness and biomechanical strength, leading to ectasia after corneal refractive surgery [14, 26]. Moreover, small-incision lenticule extraction (SMILE) removes corneal tissue, thus probably reducing the biomechanical strength of the cornea in the same way excimer laser ablation does. Therefore, it is necessary to routinely screen all patients seeking corneal refractive surgery to identify asymptomatic ectatic corneas to prevent the incidence of ectasia after corneal refractive surgery [1, 14].

In our study, the prevalence rate was calculated among patients who requested laser vision correction. Therefore, the reported prevalence rate may not represent keratoconus prevalence in the general Thai population.

Wilson et al. found that 5.7% of subjects seeking refractive surgery were classified as having definite keratoconus [27]. Talal et al. found a higher number, at 8.59% [1]. However, we found that 1.66% of patients seeking an opinion on the possibility of refractive surgery were diagnosed with keratoconus. These differences could be attributable to ethnic and geographical factors. Consanguinity might be one factor in the genetic predisposition for keratoconus. The prevalence of consanguinity is higher in the Middle East, North Africa, and South Asia. Prevalent estimates are in the ranges of 30%–50% in Middle Eastern countries, 20%–40% in North Africa, and 10%–20% in South Asia [28]. Even though there is no reported prevalence of consanguineous marriages in the Thai population, it is strongly discouraged in Thai culture. This might be the reason why the prevalence of keratoconus in Thai patients who seek refractive surgery differs from those in other populations.

Keratoconus is usually a bilateral asymmetrical disorder [29]. Keratoconus is bilateral in 69%–94.5% of cases and is unilateral in 11.8%–31% of cases [7, 9, 28, 29]. Additionally, in our study, bilateral keratoconus occurred in 97% of patients.

Regarding sex predilection, several published articles find either male or female sex to be dominant. A Mexican study reported the prevalence in females to be 2 times that of male patients (66.6% vs. 33.3%) [29]. Conversely, in a population-based study in Jerusalem, the authors reported that the prevalence in males was approximately 5 times higher than that in females (4.91% vs. 1.07%) [3]. However, some studies showed that keratoconus has no gender predominance [30, 31]. In our study, the prevalence of keratoconus in males was predominant (73.8%).

Regarding the age at the time of diagnosis, several reports showed an age ranging from 18.5 to 47.6 years. Keratoconus usually begins at puberty. According to previous studies, Asian patients showed a younger mean age at diagnosis of keratoconus [32, 33]. A study by Assiri et al. [32] in Saudi Arabia stated that the mean age at diagnosis was 18.5 ± 3.9 years. Moreover, a study by Saini et al. [33] in India showed that the mean age at diagnosis was 20.2 ± 6.4 years. In an Egyptian population study [34], the mean age at diagnosis was 29.40 ± 9.79 years and in a Macedonian study [9], the mean age at diagnosis was 26.81 ± 1.25 years. Also, in the study by Pobelle-Frasson et al. [35], the mean age of onset was 33.4 years in males and 37.1 years in females, and the study by Cruz-Becerril et al. [36] also reported that the age at onset was 28.14 ± 10.30 years [9]. However, Hashimi et al. [6] reported a mean age of 47.6 years at the time of diagnosis. In our study, the age at the time of diagnosis was 25.25 years. This, again, could be attributable to ethnicity and geographical distribution.

Most patients in the Macedonian, Saudi Arabian, Palestinian, and Malaysian studies presented with a mild disease form at the time of diagnosis [9, 30, 37, 38]. In the Macedonian study by Ljubic [9], 52.08% of patients were at the mild stage, 36.45% were at the moderate stage, and 11.57% were at the late stage. A Saudi Arabian report by Assiri et al. [32] found that 39.2% were at the mild stage, 42.5% at the moderate stage, and 18.3% at the late stage. In Shanti et al.'s study [37], 62% of the eyes were at the mild stage, 28.1% were at the moderate stage, and 9.9% were at the late stage of keratoconus. Additionally, according to a study conducted in Malaysia, 37.6%, 30.1%, 4.4%, and 27.8% of patients were at stages I, II, III, and IV, respectively, at the time of diagnosis [38].

In our study, the results were the same as in previous studies, especially those of the Malaysia study [38]. We found that 38.6%, 31.7%, 6.8%, and 22.9% of cases were at stages I, II, III, and IV, respectively, at the time of diagnosis. Most patients in the 4 studies described above had a mild to moderate form of the disease at the time of diagnosis. This finding is probably explained by several reasons [37]. First, this might be because of ophthalmologists’ awareness of keratoconus. Second, it could be an unexpected discovery during a routine check-up, arising either on account of examining the visual acuity or when changing contact lenses or glasses, because most keratoconus patients are characterized by myopic astigmatism and frequently change their glasses or contact lenses owing to the progression of their refractive errors. As in our study, 87% of the patients had new glasses prescribed because of blurred vision. Finally, the increasing trend toward laser vision correction raises the possibility of keratoconus detection.

Our study may have several limitations. As our center is a tertiary care center, the prevalence of keratoconus may be higher than the exact population-based prevalence. This may result in an exaggeration of disease severity. Moreover, the prevalence in this study is only in patients who ask for laser vision correction. However, the other demographics, the disease characteristics, and the visual parameters were not only from those who sought laser vision correction but also from those who were in the general eye and cornea clinics. Our study is the first to report this information about the Thai population.

## Conclusion

This is the first study in Thailand to report the prevalence of keratoconus among Thai patients requesting laser vision correction, which is around 1.66%. Most Thai keratoconus patients in our study were male, and presented with a mild bilateral stage of the disease in their second decade of life.
